# Isolation, self-blame and perceived invalidation in aid personnel: identifying humanitarian-specific distress using the PostAID/Q

**DOI:** 10.1186/s41018-021-00094-8

**Published:** 2021-05-03

**Authors:** Lynne McCormack, Heather Douglas, Stephen Joseph

**Affiliations:** 1 School of Psychology, Faculty of Science, University of Newcastle, Callaghan, New South Wales 2308 Australia; 2 Centre for Trauma, Resilience and Growth, School of Education, University of Nottingham, Nottingham, NG7 2RD UK

**Keywords:** PostAID/Q, Altruistic Identity/Disruption (AI/AID), Unidimensional structure, Convergent and incremental validity, Humanitarian aid personnel

## Abstract

**Objective:**

Humanitarian-specific psychological distress following deployment can elude detection using contemporary measures of trauma-related stress. This study assesses the unidimensional structure and convergent validity of the Post-deployment Altruistic Identity Disruption Questionnaire (PostAID/Q), an 18-item questionnaire underpinned by the construct *Altruistic Identity/Disruption* (*AI/AID*)*.*

**Method:**

Humanitarian aid personnel (*N*=108) completed an online web survey, inclusive of the Moral Injury Questionnaire (MIQ), Posttraumatic Distress Disorder Checklist (PCL-5), Psychological Well-Being Posttraumatic Changes Questionnaire (PWB-PTCQ) and Social Provisions Scale (SPS).

**Results:**

A confirmatory factor analysis suggested a single factor structure providing further support for the conception of AI/AID as a unidimensional construct. Convergent validity was demonstrated through (1) utility for predicting a posttraumatic stress disorder (PTSD) diagnosis assessed by the PCL-5, and (2) moral injury assessed by the MIQ. The PostAID/Q was further moderately and negatively associated with the availability of social support (assessed by the SPS) and lower self-reports of psychological well-being post trauma (assessed by the PWB-PCTQ). Finally, the PostAID/Q demonstrated evidence of incremental validity in predicting humanitarian specific psychological distress over and above the PCL-5. Specifically, the PostAID/Q predicted increased moral injury on the MIQ, and decreased psychological well-being post trauma.

**Conclusions:**

The PostAID/Q can assist in identifying humanitarian specific psychological responses post deployment guiding support for personnel, over and above more traditional measures of posttraumatic stress.

## Introduction

Humanitarian personnel are particularly susceptible to secondary traumatic stress, compassion fatigue or vicarious traumatization (Figley, 1995, 1998; McCann & Pearlman, 1990; Pearlman & Saakvitne, 1995). Such indirect exposure to a traumatic event can result in changes to memory systems, and prior views of self and the world (Figley, 1995; 1998; McCann and Pearlman 1990; Pearlman and Saakvitne 1995). Additionally, feelings of self-doubt, isolation and anxiety as well as guilt in failing to relieve the distress of the primary sufferer, can complicate the individual humanitarian’s psychological distress precipitating occupation-related health problems in the field and post-deployment (Wilson and Lindy 1994; McCormack and Joseph, 2012; McCormack et al. 2016). For example, recent attempts by aid personnel to alleviate the suffering of displaced Syrian refugees, have resulted in degraded self-differentiation, intimacy and pain associated with post trauma stress (Rizkalla and Segal 2019). This complex array of responses is not readily captured through contemporary trauma-related distress scales, leaving humanitarian organisations and their personnel with few tools to identify humanitarian-specific psychological distress post deployment. Therefore, providing a humanitarian-specific scale that more readily captures the unique impact of threat, particularly on the altruistic drive of those working in the humanitarian context, would be welcome.

Several qualitative research studies have defined the construct of *altruistic identity/altruistic identity disruption* (*AI/AID*) (McCormack et al. 2009; McCormack and Joseph 2012; McCormack et al., 2016) to explain an individual’s feeling of alienation from family, community and society on return from humanitarian work. Altruistic Identity Disruption (*AID*) is best characterised as a disruption to a healthy altruistic identity resulting in (a) inter-related feelings of isolation, doubt and self-blame; (b) questioning personal role in humanitarian work and its value; and (c) engaging in self-blame; impacting on healthy reintegration with family, career and society post-deployment. A perception that reintegration support protocols are lacking from the deploying organisation was posited as contributing to the development of these cognitive and emotional states.

As such, *AID* highlights the complexity not only of trauma exposure responses but also of the many psychosocial challenges likely faced by humanitarian personnel post deployment. For example, sector illiteracy to the specificity of humanitarian stressors can precipitate susceptibility to chronic dislocation and psychological morbidity in the aftermath of deployment including posttraumatic stress, depression and anxiety (Connorton et al. 2012). Often associated with trauma exposure symptom severity, ethical and moral conflicts that potentially arise in complex and violent humanitarian environments can precipitate a deep sense of having violated or transgressed core moral beliefs resulting in moral injury (Koenig et al. 2019). Importantly, organisational validation and support structures, in both the field and post-deployment, play a major role in personnel well-being including the provision of supervision, psychological care and team support (McCormack et al. 2016; Aldamman et al. 2019). Ironically, those experiencing *AID* often report attempting to redeploy to the field prematurely. Redeploying before any distress from prior trauma exposure has been explored, risks adding to chronic and cumulative psychological distress and further social dislocation (McCormack and Joseph 2013; McCormack et al. 2009).

The specificity of humanitarian distress as highlighted by the construct of AI/AID led to the development of the Post-deployment Altruistic Identity Disruption Questionnaire (PostAID/Q; McCormack and Joseph 2012). The conceptualization of AID as inter-related experiences all reflecting post-deployment distress suggests a one-component solution. It is important to confirm the component solution in the PostAID/Q across different samples of humanitarian aid workers, because the purpose of the scale is to provide a single tool with which to identify and make comparisons between those in need of support post-deployment. Examining the structure of the scale is important therefore to ensure that test scores based on a single-component solution are valid and useful for diagnosing and treating altruistic identity disruption (Slocum-Gori and Zumbo, 2011). In the initial study, a preliminary list of 79 items was created on which a Principal Components Analysis (PCA) was conducted (McCormack and Joseph 2012). Cattell’s (1966) scree test suggested a one-component solution, following which a forced one-component solution was computed and used to select 18 items for the final tool. Confirming the one-component solution in a second sample of humanitarian aid workers is vital to ensuring that test score interpretation of the PostAID/Q does not lead to unintended consequences from incorrectly applied treatments (Messick 1988). A further validation study conducted by McCormack et al. (2016) determined that the PostAID/Q added an additional 10% of the variation over the General Health Questionnaire-12 (GHQ-12: Goldberg and Williams 1988) in predicting the subjective response to traumatic events as measured by the Impact of Events Scale - Revised (IESR: Weiss and Marmar 1997).

It was concluded, therefore, that the PostAID/Q has the potential to assist in identifying psychosocial disruption to a healthy altruistic identity in returning aid personnel. Screening by organisations using the PostAID/Q can also assist in identifying personnel’s subsequent readiness for redeployment, by highlighting unresolved initial responses from previous humanitarian experiences creating vulnerability to chronic dislocation and psychological morbidity post-deployment. Such psychosocial readjustment is related to the duality of the complex environmental factors in the humanitarian context where risks to psychological well-being from primary and vicarious traumatization may occur, as well as the risk of burnout from cumulative exposure. The importance of organisational validation cannot be over stated for adjustment and recovery. The PostAID/Q highlights any perceived absence of validating organisational support in both the field and post-deployment.

A consequential diagnosis of posttraumatic stress disorder (PTSD) is considered likely for any humanitarian personnel following a difficult humanitarian aid experience. Therefore, if the PostAID/Q can predict additional variance, over and above PTSD symptoms, it suggests that PostAID/Q is important for predicting outcomes, developing support protocols and for assessing readiness for redeployment. Therefore, the aim of this study was to further examine the utility of the PostAID/Q as a measure of Altruistic Identity Disruption in humanitarian aid personnel. Therefore, it seeks to verify the unidimensional structure of Altruistic Identity Disruption using confirmatory factor analytic techniques, and test for its convergent validity with posttraumatic stress disorder (PTSD), as measured by the PTSD Checklist for DSM-5 (PCL-5: Weathers et al., 2013), and its incremental validity in predicting moral injury as assessed by the Moral Injury Questionnaire (MIQ: Currier et al., 2015), and psychological well-being following a traumatic event as assessed by the Psychological Well-being Posttraumatic Changes Questionnaire (PWB-PTCQ; Joseph et al., 2012). Using an online correlational design with AI/AID and subjective response to traumatic events as independent variables, and social support availability, psychological well-being posttraumatic changes, PTSD symptoms and moral injury as dependent variables, the following hypotheses were tested:

1. Confirmatory factor analysis of items on the PostAID/Q will reveal a single factor structure.
2. Altruistic identity disruption will be positively correlated with moral injury and PTSD, and negatively correlated with social support and psychological well-being.
3. Altruistic identity disruption will predict significant additional variance in moral injury and psychological well-being, over and above PTSD.

## Methods

### Participants

Participants were recruited online by emailing humanitarian organisations and Facebook Humanitarian Aid group page administrators. The email requested humanitarian organisations (e.g., Oxfam, World Vision, Red Cross/Crescent) or group administrators to promote the survey to their network. Interested parties posted the survey link to their network or Facebook. Selection criteria included fluency in English and deployment to international humanitarian work for longer than 3 months at any stage in their career with intermittent or permanent returns home.

Participants were 108 humanitarian workers who completed the online web survey. The total time these participants reported working in humanitarian aid ranged from 4 months to 17 years (204 months). On average, participants had spent 7 years and 3 months (*SD* = 4 years, 2 months) working on humanitarian aid projects. Fifteen participants (13.9%) were male and ninety-three (86.1%) reported their gender as female. Of the 104 participants who reported their age, 1 (0.9%) was under 25 years, 34 participants (31.5%) were between 25 and 34, 45 (41.7%) were between 35 and 44, 15 (13.9%) were between 45 and 54, and 9 participants (8.3%) were 55 years and older. Participants also reported how long since they returned from their last mission. A majority of participants (*N* = 51, 47.2%) reported returning home from their last mission between 0 and 3 months before. Eleven participants (10.2%) had returned 4 to 6 months previous to completing the survey, 15 (13.9%) had returned between 7 and 12 months before, and 28 (25.9%) had returned from their last mission over 12 months ago.

### Measures

#### The Post-deployment Altruistic Identity Disruption Questionnaire (PostAID/Q; McCormack and Joseph 2012)

The PostAID/Q measured altruistic identity disruption manifest by feelings of alienation, invalidation, and isolation that the participant humanitarian workers experienced after returning from humanitarian missions. It consists of 18 items responded to on a six-point Likert scale ranging from *strongly disagree* (1) to *strongly agree* (6). Example items include ‘I find it hard to feel the same about my relationships back home since aid work’. The PostAID/Q has an acceptable internal consistency reliability of *α* = .82 identified in previous research (McCormack et al. 2016).

#### The Moral Injury Questionnaire (MIQ; Currier et al. 2015)

The MIQ, a 19-item scale, assessed the moral insult experienced by individuals when required to go against their deeply held values and beliefs. Such moral insult commonly occurs in situations where individuals are exposed to trauma. Two versions of the scale have been created, one for military personnel and the other for teachers who have had a morally injurious experience. The teacher version included organisational factors relevant to humanitarian workers and was thus adopted for the current research. Respondents were asked to rate 12 *adapted* statements whilst considering their experiences as a humanitarian aid worker on a four-point Likert-type scale, ranging from *never* (1) to *often* (4). An example item from the scale is ‘I did things during deployment that betrayed my personal values’. Examination of the military version of the scale indicated a unidimensional factor structure across community and clinical samples (Currier et al., 2015).

#### The Posttraumatic Distress Disorder Checklist for DSM-5 (PCL-5; Weathers et al. 2013)

The PCL-5 is a 20-item self-report measure that assesses the 20 DSM-5 symptoms of posttraumatic stress disorder (PTSD). It replaces the earlier PCL and aligns with the new symptom cluster of PTSD in the DSM-5 (Weathers et al. 2013). Respondents were asked to think of their worst experience and indicate how much they were bothered by that experience over the past month. An example item from the scale is ‘blaming yourself or someone else for the stressful experience or what happened after it’. Participants responded on a 5-point Likert scale ranging from *not at all* (1) to *extremely* (5). The PCL-5 has demonstrated an excellent internal consistency reliability of α = .90 (Sveen et al., 2016).

#### The Psychological Well-Being Posttraumatic Changes Questionnaire (PWB-PTCQ; Joseph, Maltby and Wood et al. 2012)

The PWB-PTCQ is an 18-item questionnaire that assesses perceived changes in psychological well-being after a traumatic event. Three items assess each of the highly inter-related domains of self-acceptance, autonomy, purpose in life, relationships, sense of mastery and personal growth. Respondents were asked to rate how much they perceived themselves to have changed on each item as a result of the trauma on a five-point scale as follows; *much more so now* (5), *a bit more so now* (4), *I feel the same about this as before* (3), *a bit less so now* (2), and *much less so now* (1). Internal consistency reliability of the scale across four data collection points ranged between .87 and .95, with support found for a unidimensional factor structure (Joseph et al. 2012).

#### The Social Provisions Scale (SPS; Cutrona and Russell 1987)

The SPS is a 24-item instrument measuring the availability of social support across six domains including reassurance of worth, social integration, emotional support/attachment, tangible help, orientation, and opportunity for nurturance. An example item from this scale is ‘there are people I can depend on to help me if I really need it’. Respondents were asked to think about their current relationships with friends, family, co-workers, and the community and respond using a four-point Likert scale ranging from *strongly disagree* (1) to *strongly agree* (4). Internal consistency reliability across the six domains was variable, with Cronbach’s alphas ranging between .54 and .81 in a previous study with the PostAID/Q (McCormack et al. 2016).

### Procedure

Following human ethics approval from the University of Newcastle, Australia, participants were sourced through support networks on the web. Potential participants were invited to engage with the researchers through flyers that described the selection criteria of the study and provided information about the study. Participants who met the selection criteria received a link to Survey Monkey and were asked to complete the full questionnaire and five demographic questions online. By clicking to continue, the participants consented to participate.

### Data analyses

Missing data were examined first, to determine suitability for maximum likelihood replacement. Research with simulated datasets indicates that expectation-maximisation techniques are ideal for achieving the best possible reconstruction of sample data, regardless of sample size, proportion of missing data, and the normality of the underlying data (Gold and Bentler 2000). All scale and subscale scores were then calculated, and their distributions inspected for violations of normality.

Statistical analyses proceeded in three major stages. First, the unidimensional factor structure of the PostAID/Q was examined using confirmatory factor analytic techniques. These analyses were conducted in AMOS version 25. In contrast to common rules of thumb stating sample sizes of at least two hundred participants (Kline 2011), we used the work of Wolf et al. (2013). Wolf and colleagues conducted Monte Carlo simulations to determine sample size requirements for confirmatory factor analyses. For confirmatory factor analyses with one latent factor, at least eight indicators, and loadings of .50 or greater established by initial exploratory factor analysis (McCormack and Joseph 2012), our sample size was sufficient for achieving power ≥ .99 (Wolf et al., 2013). We used maximum likelihood estimation because the data were distributed normally (Kline 2005). The first model tested the assumption that all of the items on the PostAID/Q were subsumed by a single latent factor and that errors of measurement associated with each item were uncorrelated. This model is consistent with the theoretical formulation of altruistic identity disruption as a unitary construct, and a previous exploratory factor analysis identifying a single factor (McCormack and Joseph 2012).

In addition to the likelihood ratio test (*χ*^2^) which has some limitations in testing model fit (Jöreskog and Sörbom 1993), we reported the Comparative Fit Index (CFI; Bentler 1990), the Root Mean Square Error of Approximation (RMSEA; Browne and Cudeck 1993), and the Standardised Root Mean Square Residual (SRMR; Hu and Bentler 1995). The CFI ranges between 0 and 1, with values closer to 1 indicating better fit. The cut-off value for the CFI was originally proposed as > .90 (Bentler 1992), with a revised cut-off value of .95 more recently proposed (Hu and Bentler 1999). For the RMSEA, values less than .05 indicate good fit, and values as high as .08 represent reasonable errors of approximation in the population (Browne and Cudeck 1993). The SRMR can be interpreted as having better fit when the value is .05 or less (Byrne 2016; Hu and Bentler 1995).

Second, descriptive statistics and Cronbach’s alpha internal reliabilities were inspected, and Pearson’s correlations were computed to examine the bivariate relationships between all of the study variables. Third, to test our hypothesis regarding the incremental validity of the PostAID/Q for predicting symptoms of moral injury and psychological well-being, two hierarchical regression analyses were conducted with the MIQ and the PWB-PTCQ as dependent variables. Demographics were included in the regression analyses if they were significantly associated with PostAID/Q scores. Demographics were entered first as control variables. The PCL-5 score was entered next to control for the effect of post-traumatic stress on the two dependent variables. Finally, the PostAID/Q was entered to determine the incremental validity of the PostAID/Q over and above demographics and symptoms of post-traumatic stress. Incremental validity was evaluated by examining change in *R*^2^ values. A significant change in *R*^2^ indicates significant additional variance in the dependent variable was accounted for by the addition of the PostAID/Q. These analyses were all conducted in SPSS version 25.

## Results

Of the 108 participants who completed the survey, only 22 questions out of 12,312 (0.18% missing values) attracted no response. Only three participants had missed answering a question on the PostAID/Q (0.15% missing values on the scale). Despite the very low missing values, confirmatory factor analysis requires a complete dataset. We therefore subjected the data to a missing value analysis using the expectation-maximisation algorithm (Dempster, Laird and Rubin 1877). Little’s MCAR test identified that data was missing completely at random, *χ*^2^ (1346) = 1272.576, *p* = .923, confirming that EM estimation was appropriate for missing data replacement.

No univariate outliers were detected in any variable. All scales and subscales of the PCL-5 were slightly positively skewed, indicating that a larger portion of the sample was reporting limited disturbances associated with posttraumatic stress. A decision was taken not to transform these scales because these were valid observations in a non-clinical sample. Analyses progressed with untransformed variables in all cases.

### Confirmatory factor analysis

**Fig. 1:**
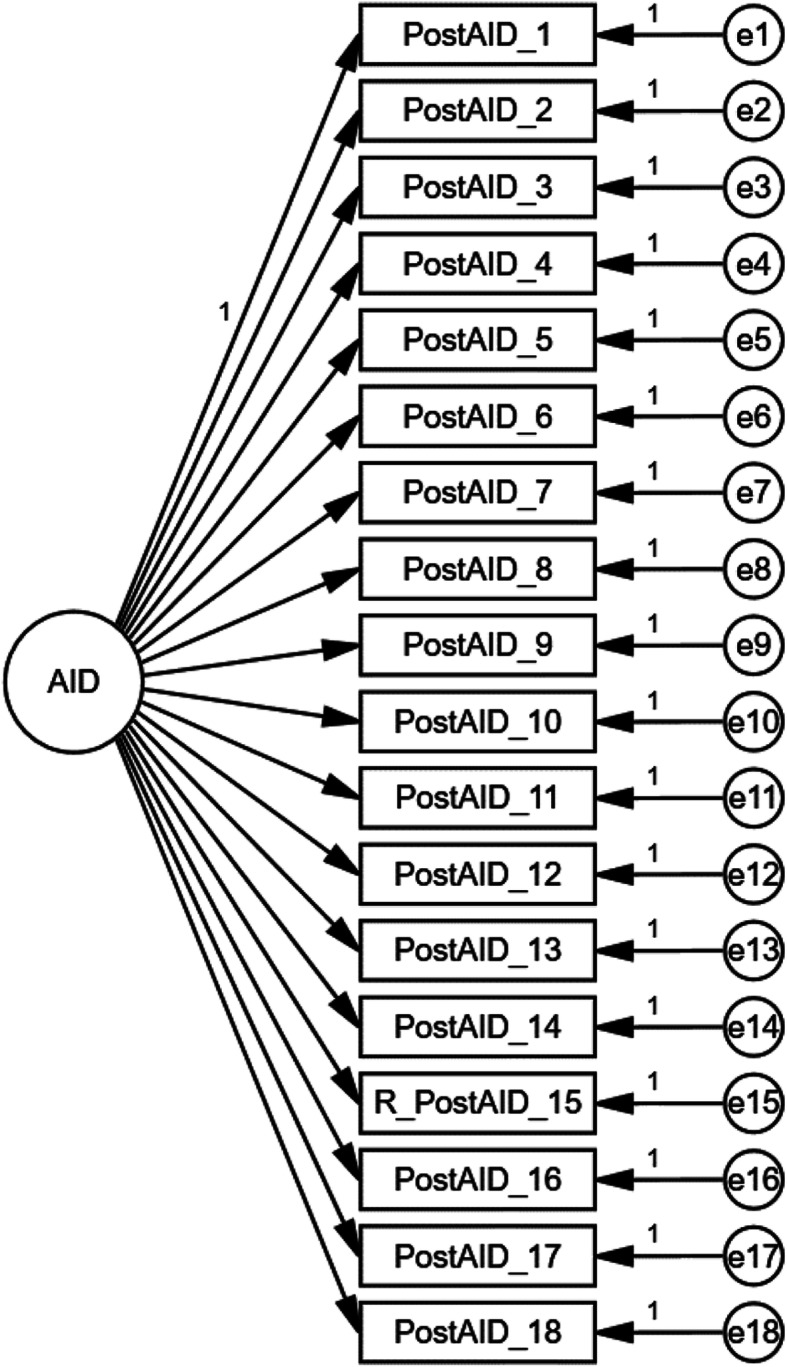
Hypothesised one-factor PostAID/Q model

Two models were tested on the PostAID/Q items. Our preferred theoretical one-factor model with uncorrelated errors can be seen in Fig. 1. The fit of each model is reported in Table 1. Based on both the likelihood ratio test and fit indices, the one-factor model with uncorrelated errors did not provide a good fit to data and was subsequently rejected. Further, both items 12 ("I found it self-reassuring if I had an emotional reaction to negative events in the field), and 17 ("I feel family members are not interested in what I did on mission") had particularly low factor loadings. This suggests that they were poor indicators of AID in the current sample.

**Table 1:** Indices for confirmatory factor analysis of the PostAID/Q

	One-factor uncorrelated errors	One-factor correlated errors	Two factor model
*χ*^2^ (df)	256.122 (135)**	178.061 (132)**	182.223 (134)*
*χ*^2^/df	1.897	1.349	1.360
CFI	.786	.919	.915
RMSEA (90% CI)	.092 (.074-.109)	.057 (.033-.078)	.058 (.034-.078)
SRMR	.0881	.0671	.0740

*p < .01

**p < .001

The second model allowed measurement errors on each item to correlate provided there was a theoretical justification for those items to be related. Modification indices on the first, uncorrelated errors model suggested that model fit would be improved by allowing three item errors to covary. Items 9, 10, and 17 all asked participants whether they feel able to share their stories about their humanitarian aid experiences with friends and family members at home. Because these items shared a consistent theme of sharing stories from their deployment with others, we allowed these errors to correlate. The model fit significantly improved and indicated a moderate to acceptable fit of a unidimensional factor structure on the PostAID/Q. The RMSEA indicated acceptable fit; however, the CFI value was between minimum acceptable fit and good fit (Hu and Bentler 1998), and the SRMR was < .10. This was not considered a major problem because a higher number of items usually lead to poorer fitting models (Floyd and Widaman 1995). This model further demonstrated a significant change in chi-square compared to the one-factor uncorrelated errors model, Δ*χ*^2^ (3) = 78.061, *p* < .001. Overall, the likelihood ratio test still indicated that this model was a significant departure from all residuals being equal to zero (Barrett 2007). As such, we tentatively interpreted the CFA model fit as providing support for the expected one-factor structure.

The one-factor correlated errors model also raised the possibility of a second latent factor on the PostAID/Q that accounted for the covariance between these three items. Subsequently, a two-factor model was examined where the three items allowed to covary were instead loaded onto a second latent factor representing the difficulty humanitarian aid workers reported in sharing their stories with friends and family at home. A change in chi-square test indicated that this model had improved fit compared to the one-factor uncorrelated errors model, Δ*χ*^2^ (1) = 73.899, *p* < .001, but not the one-factor correlated errors model, Δ*χ*^2^ (2) = 4.162, *p* = .125. Based on the theory underlying the construct of Altruistic Identity Disruption and the data currently available, we preferred the unidimensional CFA model and applied it in our further analyses. However, we cannot discount the possibility that a two-factor model fits just as well as the unidimensional account of altruistic identity disruption. Both standardised and unstandardised estimates are provided in Table 2.

**Table 2:** Standardised and unstandardised coefficients for PostAID/Q with correlated errors

Path	*β*	*B*	SE	CR	SMC
PostAID_1 ← AID	.595	1			.354
PostAID_2 ← AID	.464	.836	.201	4.157*	.215
PostAID_3 ← AID	.733	1.484	.252	5.897*	.537
PostAID_4 ← AID	.669	1.193	.215	5.537*	.448
PostAID_5 ← AID	.768	1.623	.267	6.078*	.589
PostAID_6 ← AID	.546	1.053	.222	4.753*	.298
PostAID_7 ← AID	.520	.877	.192	4.567*	.270
PostAID_8 ← AID	.525	.984	.214	4.606*	.276
PostAID_9 ← AID	.401	.727	.198	3.672*	.161
PostAID_10 ← AID	.376	.597	.172	3.466*	.141
PostAID_11 ← AID	.509	.822	.183	4.492*	.259
PostAID_12 ← AID	.132	.246	.192	1.277	.017
PostAID_13 ← AID	.543	1.103	.233	4.727*	.294
PostAID_14 ← AID	.587	.960	.191	5.024*	.344
R_PostAID_15 ← AID	.608	.952	.184	5.164*	.370
PostAID_16 ← AID	.368	.678	.199	3.401	.135
PostAID_17 ← AID	.211	.414	.205	2.024*	.045
PostAID_18 ← AID	.652	1.114	.205	5.438*	.426
e10 ↔ e17	.569	1.156	.229	5.046*	
e9 ↔ e10	.513	.902	.196	4.600*	
e9 ↔ e17	.468	1.072	.248	4.314*	

*SE* standard error, *CR* critical ratio, *SMC* squared multiple correlation

Note: PostAID_1 was set as the reference variable

* = *p* < .05

### Descriptive statistics

Descriptive statistics, internal reliabilities, and bivariate correlations between study variables can be found in Table 3. Internal consistency reliabilities were acceptable for all scales including the PostAID/Q. There were no differences in PostAID/Q scores by gender, *t* (106) = −.634, *p* = .527, age, *F* (5, 102) = 1.599, *p* = .167, or time since return from humanitarian mission, *F* (4, 103) = .203, *p* = .936. Total time spent in humanitarian aid work was positively associated with the PostAID/Q, the PCL5 and the MIQ. Time in aid was controlled for in subsequent multivariate analyses.

**Table 3:** Descriptive statistics, internal reliability and correlations between study variables including all subscales

	M	SD	1	2	3	4	5	6	7	8	13	14	15	16	17	18	19	20
1. Time in aid	87	50	**n/a**															
2. PostAIDQ	67.79	15.48	.20*	**.87**														
3. PCL5	20.5	17.98	.21*	.60**	**.96**													
4. PCL5_Intrusions	4.14	4.49	.19	.49**	.88**	**.89**												
5. PCL5_Avoidance	2.04	2.19	.20*	.55**	.86**	.73**	**.84**											
6. PCL5_NACM	7.79	7.00	.15	.58**	.96**	.80**	.81**	**.91**										
7. PCL5_AR	6.55	5.71	.23*	.57**	.94**	.79**	.75**	.85**	**.85**									
8. MIQ	30.48	5.95	.21*	.51**	.44**	.38**	.39**	.41**	.44**	**.78**								
9. PWB_PTCQ	60.58	13.25	−.14	−.44**	−.50**	−.40**	−.38**	−.50**	−.47**	−.16	**.93**							
10. SPS	74.85	10.6	−.09	−.41**	−.47**	−.37**	−.35**	−.48**	−.43**	−.22*	.46**	**.92**						
11. SPS_Guidance	12.8	2.36	−.15	−.34**	−.42**	−.36**	−.36**	−.40**	−.40**	−.21*	.42**	.85**	**.87**					
12. SPS_ReassuranceofWorth	12.64	1.97	−.06	−.42**	−.41**	−.29**	−.34**	−.40.**	−.37**	−.20*	.40**	.68**	.55**	**.74**				
13. SPS_SocialIntegration	12.26	2.34	−.11	−.30**	−.39**	−.26**	−.27**	−.41**	−.35**	−.10	.44**	.80**	.58**	.55**	**.82**			
14. SPS_Attachment	12.15	2.57	−.08	−.40**	−.37**	−.27**	−.23*	−.40**	−.35**	−.18	.39**	.86**	.70**	.44**	.65**	**.76**		
15. SPS_Nurturance	11.67	2.46	.17	−.07	−.10	−.11	−.04	−.12	−.07	−.03	.05	.50**	.25**	.13	.32**	.32**	**.76**	
16. SPS_ReliableAlliance	13.33	2.31	−.18	−.35**	−.46**	−.39**	−.36**	−.44**	−.44**	−.26**	.41**	.83**	.78**	.52**	.55**	.73**	.20*	**.87**

*PCL5* Posttraumatic Stress Disorder Checklist, *NACM* negative alterations in cognitions and mood, *AR* alterations in arousal and reactivity, *MIQ* Moral Injury Questionnaire, *PWB-PTCQ* Psychological Well-Being-Posttraumatic Changes Questionnaire, *SPS* Social Provisions Survey

Note: Cronbach’s alpha internal reliabilities reported in bold on the diagonal

^*^p < .05

^**^ *p* < .01

### Convergent validity

The PostAID/Q was positively and strongly associated with the PCL5 and the MIQ, indicating that increasing self-reported altruistic identity disruption was positively associated with self-reports of posttraumatic stress disorder and moral injury. The PostAID/Q also was moderately and negatively related to PWB and SPS, indicating that increasing levels of altruistic identity disruption were associated with decreasing levels of psychological well-being following traumatic events and lower levels of self-reported social support. A full correlation table showing associations between all measurement subscales can be found in Table 3.

### Incremental validity

Two hierarchical linear regressions were run to assess the incremental validity of the PostAID/Q for predicting variance in MIQ and PWB-PTCQ scores. Time spent working in humanitarian aid was added first, followed by scores on the PCL-5, then the PostAID/Q. No multivariate outliers were detected and all assumptions of normality, linearity, homoscedasticity and an absence of multicollinearity were met (Tabachnick and Fidell 2013). Results of the regression analyses are reported in Table 4.

**Table 4:** Hierarchical regression analysis of predictors of psychological well-being and moral injury

	Step 1 (PCL-5)				Step 2 (PostAID/Q)			
	*B*	SE	*β*	*t*	*B*	SE	*β*	*t*
**PWB-PTCQ**								
Time in aid	−.01	.02	−.04	−.40	−.01	.02	−.02	−.23
PCL-5	−.37	.06	−.50	−5.95**	−.27	.08	−.37	−3.56**
PostAID/Q					−.19	.09	−.22	−2.15*
*R*^2^	.25				.28			
Δ *R*^2^					.03*			
**MIQ**								
Time in aid	.01	.01	.12	1.32	.01	.01	.09	1.06
PCL-5	.15	.03	.44	5.02**	.07	.03	.20	1.99*
PostAID/Q					.15	.04	.39	3.81**
*R*^2^	.19				.29			
Δ *R*^2^					.10**			

*p < .05

**p < .001

In the first hierarchical regression, the PCL-5 scores accounted for 22.9% of the variance in PWB-PTCQ scores over and above time spent in humanitarian aid work (Δ*R*^2^ = .229, *p* < .001). Adding the PostAID/Q to the model explained an additional 3.1% of the variation in PWB-PTCQ scores. This was a statistically significant improvement, Δ*R*^2^ = .031, *p* = .043. However, 3.1% additional variance accounted for is not likely to suggest clinical utility for the PostAID-Q. The second hierarchical regression examined moral injury as the dependent variable. The PCL-5 scores accounted for 16.3% of the variance in MIQ scores after accounting for time spent in humanitarian work (Δ*R*^2^ = .163, *p* < .001). The addition of the PostAID/Q explained an additional 10.4% of the variance in moral injury. This was a significant change, Δ*R*^2^ = .104, *p* < .001.

## Discussion

The PostAID/Q is a tool for identifying distress in humanitarian aid workers that goes beyond traditional conceptions of posttraumatic stress disorder. Such a tool must satisfy rigorous psychometric criteria to have utility in providing post-mission care to humanitarian aid workers (Haynes and Lench 2003). Consistent with the theoretical underpinnings of the altruistic identity disruption construct, our first hypothesis was that a unidimensional factor structure would fit items from the PostAID/Q well. Confirmatory factor analyses revealed that the PostAID/Q was reasonably accounted for by a unidimensional, correlated errors model. However, two items did not load well onto the unidimensional factor structure. Item 12 in particular sought positive self-recognition of distress and was therefore a poor indicator of altruistic identity disruption while the other items on the scale represented doubts, difficulty coping, and challenges sharing stories with others. While the unidimensional correlation errors model did account for the data, other models represented the data equally well, with the two-factor model incorporating a second factor reflecting a perceived isolation of the individual from their friends and family provided a similar statistical fit to the data. The description of the items loading on this unexpected second factor seems consistent with the description of AID as, at least in part, feelings of isolation. Further, two-factor confirmatory models require a sample size of at least 150 to achieve 80% power (Wolf et al. 2013), a sample size that we did not achieve. Whilst these findings are consistent with previous exploratory factor analyses conducted by McCormack and Joseph (2012), who also uncovered a single factor structure, we cannot discount the possibility that a two-factor model might account for the data better. It is possible that our findings suggest that AID is in fact a multi-dimensional construct, with at least one factor representing the experiences of isolation from friends and family. Findings from the current study provide inconclusive support for the conception of AI/AID as a unidimensional construct, and require further testing with more robust sample sizes.

In support for the convergent validity of the PostAID/Q, bivariate correlations revealed that the AID construct was strongly and positively correlated with the potential for posttraumatic stress disorder symptoms assessed by the PCL-5, and moral injury assessed by the MIQ. This supports our suggestion that altruistic identity disruption is a useful construct to describe experiences in the wake of being involved in humanitarian traumatic events. Though we would expect perceived threat to increase an individual’s distress levels, whether that is threat to self, moral threat, or disruption to altruistic identity, the precipitating event that results in higher scores for reported post-traumatic stress, moral injury and/or altruistic identity disruption vary and uniquely predict the consequential distress that will impact the individual. The PostAID/Q was further moderately and negatively associated with the perceived availability of social support (assessed by the SPS) and psychological well-being after posttraumatic changes. The findings on the SPS are consistent with the findings of McCormack et al. (2016) using the same scale. As such, individuals who scored high in altruistic identity disruption reported feeling more isolated, less supported by their organisation post deployment, and were inclined to self-blame. This indicates that the PostAID/Q readily identifies interrelated responses specific to altruistic identity disruption not identified in other distress scales, and is therefore useful for assessing humanitarian specific distress post deployment, and readiness for redeployment.

The final hypothesis of this study was that dealing with incremental validity. The PostAID/Q supported our general hypothesis, demonstrating evidence of incremental validity over and above the PCL-5 in increasing levels of moral injury on the MIQ. The PostAID/Q predicted an additional 10.4% variance on the MIQ. This suggests that Altruistic Identity Disruption is an important additional distress construct that could be useful for assessing humanitarian aid worker distress, over and above more traditional measures of posttraumatic stress. However, the PostAID/Q was only able to predict an additional 3.1% variance on the PWB-PTCQ, suggesting that it may be less successful in understanding positive psychological outcomes following humanitarian-specific traumatic events. Relative to other measures, it appears that the PostAID/Q gives significant additional information on the psychosocial distress experienced by humanitarian workers returning from aid missions.

### Limitations and future research directions

The sample size of one hundred and eight humanitarian workers might be considered small for a cross-sectional, correlational study of this nature. However, two arguments speak against such a criticism of this study. The first is the large correlations observed between study variables. Using the smallest bivariate correlation observed between the PostAID/Q and the Social Provisions Scale (SPS), post hoc power analyses revealed that the current study had 99% power to detect such an effect size with an alpha level of .05. Second, for the incremental validity test with the MIQ as the dependent variable, the post hoc power for a multiple regression testing the change in *R*^2^ associated with the PostAID/Q was .91. Both of these post hoc power calculations speak to the sufficiency of the sample size in this study.

Several studies now highlight the utility of the PostAID/Q for guiding humanitarian organisations in the psychosocial aftercare of personnel and subsequent readiness for redeployment by accessing broader re-integration distress particularly perceived organisational support related to events in the field. Collaborative use of the PostAID/Q scores between returning personnel and their organisation is recommended to (a) normalise post deployment humanitarian-specific distress responses; (b) seek and value feedback; (c) educate and assist family on the psychosocial processes of reintegration following humanitarian deployment and (d) monitor and support personnel during the reintegration and redeployment stages. Whilst the current study comprehensively assessed the convergent and incremental validity of the PostAID/Q, the divergent validity of the scale was not examined. A reasonable test of divergent validity might examine the overlap of the PostAID/Q with other measures of depression, anxiety or psychological distress not captured in these studies. Testing the utility of PostAID/Q in national volunteers, and other front-line career groups such as military personnel returning from deployment, is recommended.

## Data Availability

The datasets generated and/or analysed during the current study are not publicly available due to accessibility but are available from the corresponding author on reasonable request.
